# ACAD10 protein expression and Neurobehavioral assessment of *Acad10*-deficient mice

**DOI:** 10.1371/journal.pone.0242445

**Published:** 2020-12-10

**Authors:** Kaitlyn Bloom, Anuradha Karunanidhi, Kimimasa Tobita, Charles Hoppel, Edda Thiels, Eloise Peet, Yudong Wang, Shrabani Basu, Jerry Vockley

**Affiliations:** 1 School of Medicine, University of Pittsburgh, Pittsburgh, Pennsylvania, United States of America; 2 School of Medicine, Case Western Reserve University, Cleveland, Ohio, United States of America; 3 Department of Neurobiology, University of Pittsburgh, Pittsburgh, Pennsylvania, United States of America; 4 Center for the Neural Basis of Cognition, University of Pittsburgh, Pittsburgh, Pennsylvania, United States of America; 5 Center for Rare Disease Therapy, UPMC Children’s Hospital of Pittsburgh, Pittsburgh, Pennsylvania, United States of America; 6 Graduate School of Public Health, University of Pittsburgh, Pittsburgh, Pennsylvania, United States of America; UAB School of Medicine, UNITED STATES

## Abstract

Acyl-CoA dehydrogenase 10 (*Acad10*)-deficient mice develop impaired glucose tolerance, peripheral insulin resistance, and abnormal weight gain. In addition, they exhibit biochemical features of deficiencies of fatty acid oxidation, such as accumulation of metabolites consistent with abnormal mitochondrial energy metabolism and fasting induced rhabdomyolysis. ACAD10 has significant expression in mouse brain, unlike other acyl-CoA dehydrogenases (ACADs) involved in fatty acid oxidation. The presence of ACAD10 in human tissues was determined using immunohistochemical staining. To characterize the effect of ACAD10 deficiency on the brain, micro-MRI and neurobehavioral evaluations were performed. *Acad*10-deficient mouse behavior was examined using open field testing and DigiGait analysis for changes in general activity as well as indices of gait, respectively. ACAD10 protein was shown to colocalize to mitochondria and peroxisomes in lung, muscle, kidney, and pancreas human tissue. *Acad10*-deficient mice demonstrated subtle behavioral abnormalities, which included reduced activity and increased time in the arena perimeter in the open field test. Mutant animals exhibited brake and propulsion metrics similar to those of control animals, which indicates normal balance, stability of gait, and the absence of significant motor impairment. The lack of evidence for motor impairment combined with avoidance of the center of an open field arena and reduced vertical and horizontal exploration are consistent with a phenotype characterized by elevated anxiety. These results implicate ACAD10 function in normal mouse behavior, which suggests a novel role for ACAD10 in brain metabolism.

## Introduction

Mitochondrial β-oxidation of fatty acids is critical for maintaining energy balance in mammals. Fatty acids are also essential components of complex lipids including those critical to membrane structure. Fats involved in energy storage (typically 16 and 18 carbon species), undergo a series of enzymatic reactions in the mitochondria called fatty acid β-oxidation (FAO) when an organism needs additional energy during times of fasting or physiologic stress. FAO involves a series of linked substrate transporters and enzymes ultimately leading to the release of the acetyl-CoA for the tricarboxylic acid cycle (TCA, also known as the Krebs cycle), along with reducing equivalents that channel directly to the electron transport chain (ETC) [1]. The end result is an increase in the production of mitochondrial ATP [2]. Acyl-CoA dehydrogenases (ACADs) catalyze the α,β-dehydrogenation of acyl-CoA esters in the first intra-mitochondrial step of FAO, utilizing an essential flavin adenine dinucleotide (FAD) cofactor to oxidize the substrate [3]. Several acyl-CoA dehydrogenases function in fatty acid oxidation, each with a unique substrate optimum based on carbon chain length. Four additional ACADs have been described that are active in the metabolism of branched chain amino acids and lysine.

We have recently identified and characterized the function of another protein annotated in the human genome and predicted to be a mitochondrial acyl-CoA dehydrogenase (ACAD10) [4, 5]. The *ACAD10* gene is located within a complicated locus containing multiple predicted exons and protein domains, including an ACAD domain. ACAD10 protein expression has been reported to be enhanced following downregulation of mechanistic target of rapamycin 1 (mTORC1) or reduced expression of mitochondrial complexes [6]. *ACAD10* is highly expressed in fetal brain [7, 8]. Previous studies have suggested potential functions for ACAD10 protein in the central nervous system, metabolism, and immunity, but no functional studies were performed [7]. A randomized clinical trial has identified an association of an *ACAD10* variant with neovascular age-related macular degeneration (AMD) [9]. We recently have characterized an *Acad10* gene trap knockout mouse model [7]. Deficient mice exhibited a blood acylcarnitine profile in older animals consistent with a disruption of long-chain FAO, developed fasting rhabdomyolysis, and had abnormal skeletal muscle mitochondria. Surprisingly, *Acad10*-deficient mice accumulated excess abdominal adipose tissue, an early inflammatory liver process, and developed late-onset insulin-resistant diabetes mellitus [7]. An unrelated study to evaluate long non-coding RNA (lncRNA) expression found that ACAD10 may be necessary for proper regulation of brown and white adipose tissue [10]. To understand the neurologic role of ACAD10 in intermediary metabolism and its association with the brain, neurobehavioral assessment was performed on wild-type and *Acad10*-deficient mice utilizing the open field test. Detailed analysis of their gait was performed to assess movement abnormalities, such as those associated with motor function-related neurologic disorders [11–14]. Additional histologic and functional imaging of *Acad10*-deficient mouse brain was performed.

## Materials and methods

### *Acad10* gene trap knockout mouse model

*Acad10*-deficient mice have previously been described [7]. Wild-type mice with the same 129SvEv/BL6 mixed background were bred as controls. Genotype was confirmed using genomic DNA in a PCR reaction. PCR products were detected using agarose gel electrophoresis. All mouse experiments were approved by the University of Pittsburgh’s Institutional Animal Care and Use Committee.

### Subcellular location of FAO and respiratory chain proteins

A western blot survey of FAO and respiratory chain protein expression in mouse heart, liver, muscle, and brain was performed by homogenizing approximately 100 mg of tissue in ice cold PBS using a Virtis HandiShear (The Virtis Company, Gardinier, New York) homogenizer followed by sonication using a microtip, and centrifugation at 30 minutes, 14,000 x g, at 4°C. The supernatant (100 μg protein) was loaded onto an SDS-PAGE gel and transferred to Immun-Blot PDVF membrane (BioRad, Hercules, California). MitoProfile Total OXPHOS Rodent WB antibody cocktail (Abcam, Cambridge, MA), citrate synthase (C-20) (Santa Cruz Biotech Inc, Dallas, TX), VLCAD, and MCAD were used for visualization of western blots of 4–15% SDS polyacrylamide gels.

### Immunohistochemistry

A human tissue panel was obtained from the NIH’s Eastern Division of the Cooperative Human Tissue Network (CHTNED). Formalin Fixed Paraffin Embedded (FFPE) human tissues were sectioned to 5 μm thickness. The sections were deparaffinized, rehydrated, and subjected to antigen retrieval followed by cell permeablization, then briefly washed with PBS and blocked. Tissues were stained by overnight incubation with three primary antibodies: anti-ACAD10, anti-cytochrome c oxidase subunit 4, and anti-catalase. ACAD10 purified antibody was used in a 1:1000 dilution and anti-MTCO1 antibody as a mitochondrial marker (Abcam), and catalase (N-17) (Santa Cruz Biotechnology, Inc.) were used in 1:250 dilutions for the lung. Two different antibody dilutions were used to obtain the best possible tissue images. ACAD10 purified antibody was used in a 1:500 dilution, and anti-MTCO1 antibody as a mitochondrial marker (Abcam), and catalase (N-17) (Santa Cruz Biotechnology, Inc.) were used in 1:250 dilutions for kidney, liver, muscle, and pancreas. After washing, sections were further incubated for 1 hour with secondary antibodies donkey anti-rabbit 488 for ACAD10, donkey anti-mouse Cy3 for mitochondria, and donkey anti goat Cy5. Nuclei were counterstained with DAPI. Aqueous anti-fade fluorescent mounting medium was applied and imaged. Slides were analyzed using an Olympus FluoView FV1000 confocal microscope (Olympus).

### Biochemical analyses in *Acad10*-deficient mouse brain and plasma

Mouse brain and plasma were collected and flash frozen in liquid nitrogen, then extracted and derivatized with pentafluorophenacyl trifluoromethanesulfonate. Acylcarntine profiling using ultra-high-performance liquid chromatography tandem mass spectroscopy (UHPLC MS/MS) was performed, as previously described [15]. Analyses were performed in triplicate and values were quantified using 13-point calibration curves.

### Micro-MRI imaging

Brain MRI was performed on wild-type and *Acad10*-deficient mice to examine brain structure in the UPMC Children’s Hospital of University of Pittsburgh animal imaging facility using a micro-MRI system (Horizontal bore 7-T MRI system, Bruker Biospin 70/30 (Bruker, Billerica, MA) with full vital monitoring. Mice were anesthetized using isoflurane with 100% oxygen gas via nose cone during MRI imaging. Mouse body temperature, heart rate, respiratory rate, and arterial oxygen saturation were continuously monitored using a real-time physiology monitoring system. Animals were also visually monitored during the imaging. Total scanning time was approximately 30 to 45 minutes per mouse.

### Open field testing

Open field testing in mice was performed using standard protocols [16]. Briefly, to examine general activity in an open environment that at the start of the test was novel to the animals, a total of 6–7 *Acad10*-deficient mice and 5–6 wild-type mice were tested at least 5 months prior to excess weight gain. After an acclimatization period of 10–15 minutes in the behavioral testing room, mice were placed individually into one of the four corners of an open-field testing chamber (28 x 28 x 40 cm; Med Associated Inc., St. Albany, VT) and allowed to move freely in the test arena for 30 minutes. Mice were tested between the hours of 09:00 and 14:00 in random order with respect to genotype. Lighting was set to 15–20 lux, similar to the light level in the colony room. The following measures were analyzed: (1) distance and (2) time of ambulation, (3) counts and (4) time of movement while stationary (e.g., grooming), (5) resting time, and (6) counts and (7) time of rearing. These measures were assessed in the center of the test arena and, separately, for the space adjacent to the chamber walls, i.e., the surround. Results were combined for the center and surround unless otherwise noted and compared between genotypes using two-tailed Student’s T-tests for independent groups and applying Welch’s correction for unequal variances where appropriate. The significance level was set to α ≤ 0.05.

### DigiGait testing

A ventral plane treadmill videographic system (DigiGait; Mouse Specifics, Inc., Framingham, MA) was used to image the ventral view of animals as they walked on a motorized transparent treadmill belt. A total of 4 *Acad10*-deficient mice and 3 wild-type mice were tested at least 5 months prior to excess weight gain. After an acclimatization period of 5 minutes in the behavioral testing room, mice were placed individually on a motorized treadmill with a transparent treadmill belt. At the start of each daily trial, the motor was idle and mice were allowed to familiarize themselves with the test apparatus for 10 minutes. On Day 1, testing entailed the familiarization period only. On Days 2 to 4, the belt speed was increased gradually 5 cm/s on Day 2, from 5 cm/s to 10 cm/s and then to 15 cm/s on Day 3, to approximately 29 cm/s on Day 4. Mice were imaged from underneath with a high-speed digital video camera for at least 30 seconds, to capture a minimum of 8 complete ambulatory cycles. Data captured on Day 4 were used for data analysis.

Lights in the room were turned off, except for the lighting fixtures in the chamber. DigiGait proprietary software was used to analyze the images. One clip of data was analyzed for each animal, and parameters for best visualization were set for each animal. The experimenter carrying out the analysis was blinded to the genotype of the mice. The following measures were analyzed statistically: (1) braking time (duration of the braking phase), (2) brake stance (percentage of the stance phase that the paw is in the braking phase), (3) propulsion time (duration of the propulsion phase), (4) propel stance (percentage of the stance phase that the paw is in the propulsion phase), (5) stride length, (6) stride frequency, (7) stance time (duration of the stance), and (8) swing (time duration of the swing phase). For each of these measures, an average value was generated for each animal from 8 complete ambulatory cycles. Results were compared between genotypes using two-tailed Student’s T-tests for independent groups and applying Welch’s correction for unequal variances where appropriate. The significance level was set to α ≤ 0.05.

All behavioral testing was carried out in the Rodent Behavior Analysis Core facility at the University of Pittsburgh School of Health Sciences.

## Results

### FAO and ETC complex protein expression

**Fig 1:**
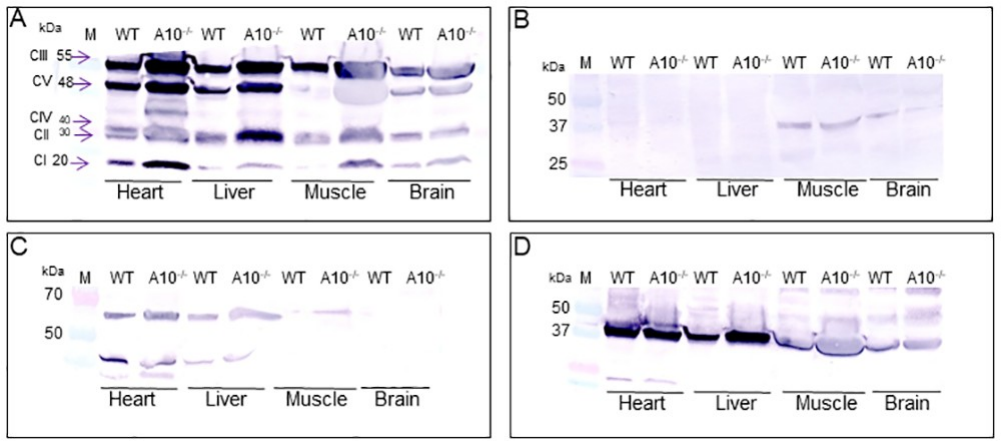
Western blot survey of ETC subunits and ACADs in mouse heart, liver, muscle, and brain. A 4–15% SDS-PAGE gel with 50 μg of tissue extract applied per lane was visualized by western blotting with antiserum to the indicated protein. (A) Mitochondrial protein subunits complex III (55 kDa), complex V (48 kDa), complex IV (40 kDa), complex II (30 kDa), and complex I (20 kDa) were visualized in heart, liver, muscle, and brain using a mitochondrial cocktail antibody. (B) Immunostaining for citrate synthase revealed a 37 kDa band. Western blots using anti VLCAD (C) and MCAD (D) antisera showed the appropriate protein sizes of 62 kDa and 42 kDa, respectively.

**Fig 2:**
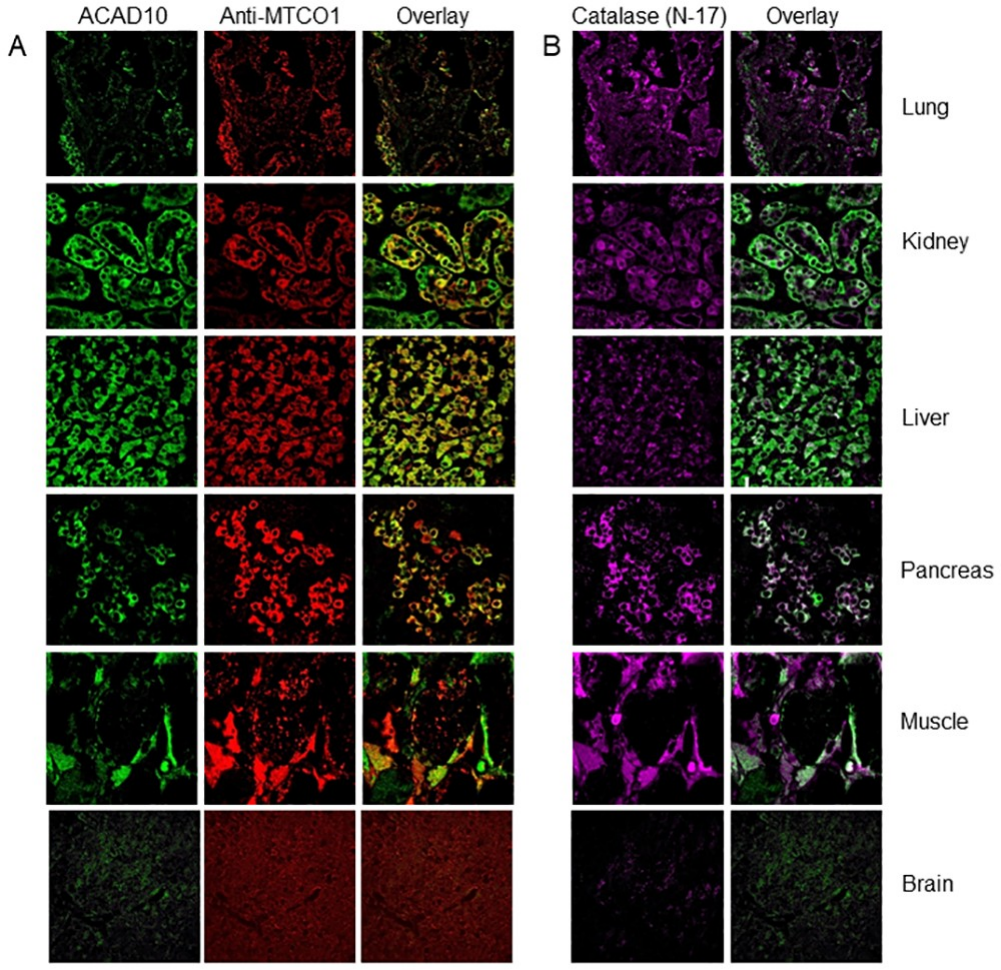
Immunofluorescent staining of lung, kidney, liver, pancreas, muscle, and brain from human tissues. (A) Immunofluorescent staining of lung, kidney, liver, pancreas, muscle, and brain from human tissues with antiserum to ACAD10 (left column), the mitochondrial marker MTCO1 (middle column), and the merged image (right column). Lung, pancreas, muscle, and kidney showed co-localization of ACAD10 with the mitochondrial marker. (B) In addition, human tissues were stained with antibodies to the peroxisomal marker catalase (left column). The right column shows the merged images and identifies colocalization of ACAD10 to peroxisomes as well as mitochondria in lung and pancreas. Slides were analyzed using an Olympus FluoView FV1000 confocal microscope with a magnification of 60X.

ETC complex activity was previously found to be elevated in liver, muscle, and brain from *Acad10*-deficient animals, suggestive of mitochondrial proliferation [7]. To further evaluate this earlier finding, mitochondrial respiratory chain subunits and FAO proteins were assessed by SDS-PAGE in tissue extracts from wild-type and *Acad10*-deficient mouse tissues. Mitochondrial respiratory chain subunits were detected in all tissues at the appropriate molecular mass and were minimally increased by loss of ACAD10 (Fig 1A). While this finding could suggest mitochondrial proliferation, citrate synthase, a typical marker of this phenomenon was present at similar levels in muscle tissue and minimally detected or not identifiable in other tissues (Fig 1B). Additionally, VLCAD and MCAD were observed in heart, liver, and muscle (Fig 1C and 1D, respectively) were present in similar amounts in both control and *Acad10*-deficient animals. In sum, these findings do not support a significant mitochondrial presentation in mutant mice. Immunohistochemical staining of a variety of wild-type mouse tissues using ACAD10 antibodies was previously reported [7]. Immunohistochemical staining of additional human tissues using ACAD10 antibodies is shown in Fig 2 and supports the localization of ACAD10 to one or both organelles in various human tissues.

### Biochemical analyses of *Acad10*-deficient mouse brain and plasma

A variable pattern of elevated long-branch-chained acylcarnitines was observed in both brain extracts and plasma from *Acad10*-deficient animals (Table 1). This pattern of elevated metabolites failed to suggest an exact enzymatic function of ACAD10. A complete table of acylcarnitine species measured is shown in S1 Table.

**Table 1:** Acylcarnitines in wild-type and *Acad10*-deficient mouse brain and plasma.

	Brain		Plasma	
Acylcarnitine species	Wild-type (mean ± SD)	ACAD10-/- (mean ± SD)	Wild-type (mean ± SD)	ACAD10-/- (mean ± SD)
valerylcarnitine	0.148 ± 0.11	0.163 ± 0.15	0.008 ± 0.01	0.016 ± 0.01
isovalerylcarnitine	0.644 ± 0.22	0.789 ± 0.62	0.107 ± 0.06	0.13 ± 0.06
S-3-hydroxy-hexanoylcarnitine	0.116 ± 0.12	0.125 ± 0.14	0.001 ± 0.00	0.005 ± 0.01
trans-2-tetradecenoylcarnitine	0.199 ± 0.29	0.23 ± 0.23	0.004 ± 0.01	0.007 ± 0.01
S-3-hydroxy-myristoylcarnitine	0.083 ± 0.13	0.093 ± 0.14	0.004 ± 0.01	0.007 ± 0.01
gamma-linolenoylcarnitine	0.114 ± 0.18	0.120 ± 0.21	0.004 ± 0.01	0.007 ± 0.01
succinylcarnitine	0.283 ± 0.13	0.299 ± 0.03	0.024 ± 0.01	0.032 ± 0.02

Table shows means ± standard deviation.

### Micro-MRI structural imaging of *Acad10*-deficient mouse brain

**Fig 3:**
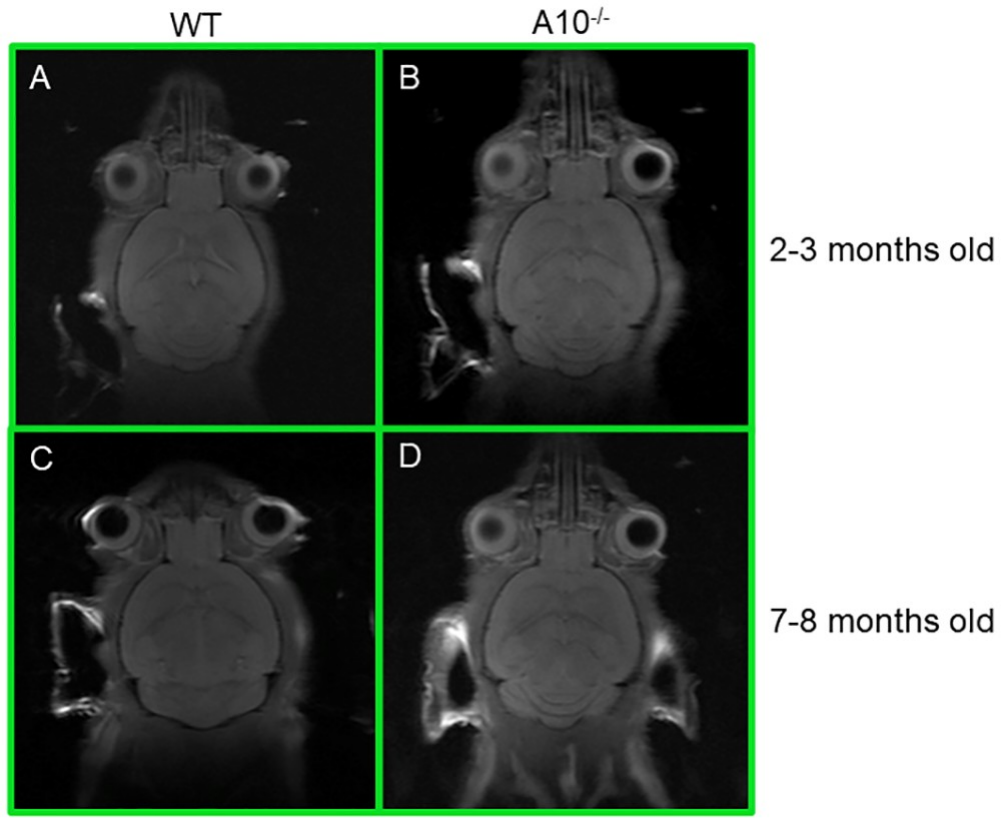
Micro-MRI of mouse brain. Mice were visualized using Horizontal bore 7-T MRI system, Bruker Biospin 70/30 with full vital monitoring system. (A) and (B) is the same wild type mouse at 2–3 months old and 7–8 months old, respectively, using RARE-T1 Imaging. The coronal image is whole, live, mouse brain section 11 of 15 sections. (C) and (D) is the same *Acad10*-deficient mouse at 2–3 months old and 7–8 months old, respectively, using RARE-T1 Imaging. The coronal image is a representative whole, live, mouse brain section 11 of 15 sections. The *Acad10*-deficient mice do not show any significant changes in brain structure and morphology over time as compared to wild type control mice of the same background.

*Acad10*-deficient mice showed no structural abnormalities of the brain at 2–3 months of age with micro-MRI scanning (Fig 3). Because they ultimately proved to have subtle abnormalities on neurobehavioral testing, additional brain MRI scans were performed up to 8 months of age to identify any changes in morphology over time. MRI images were gathered using software for RARE-T1 imaging. All images shown in Fig 3 are of brain slice 11 out of 15 slices, and, as a result, are specifically matched. No changes were identified in brain structure (Fig 3).

### Neurobehavioral open-field testing

**Fig 4:**
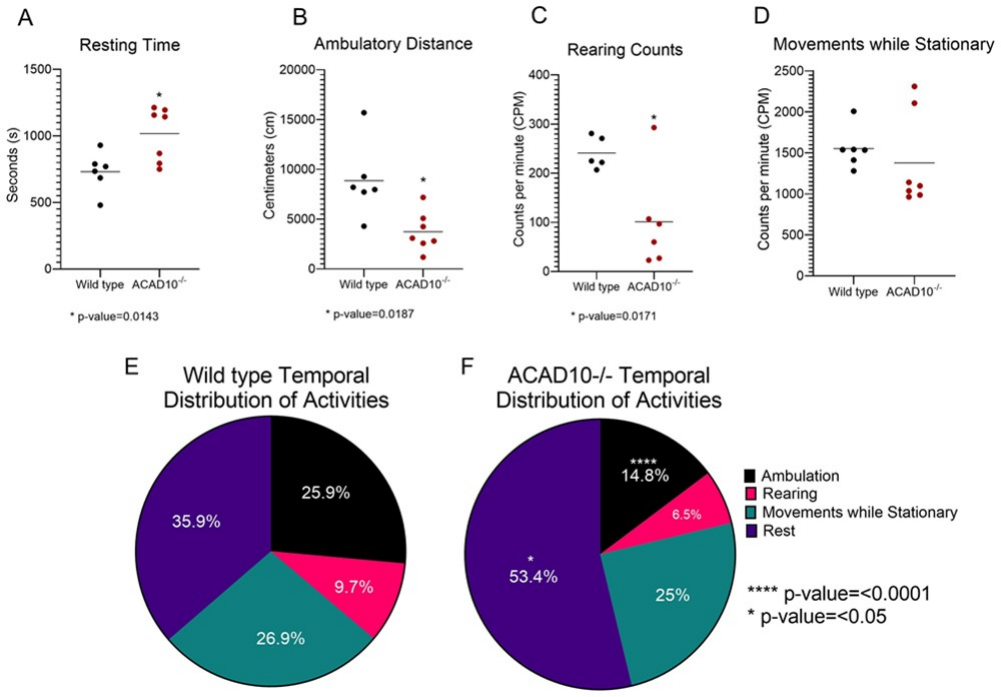
Open field measures for wild-type and *Acad10-*deficient mice. (A-D) Group averages (horizontal lines) of rest time, distance travelled, counts per minute (CPM) of rearing, and CPM of movements while stationary for wild-type (black dots) and *Acad10*-deficient mice (red dots). During the 30-minute test, *Acad10*-deficient animals spent statistically significantly more time resting (A), travelled a statistically significantly shorter distance (B), and reared statistically significantly less frequently (C) compared to wild-type mice of the same background (unpaired Student’s T-test with Welch’s correction, p-value <0.05 to < 0.0005). The frequency of movements while stationary (D) did not differ significantly between *Acad10*-deficient mice and wild-type mice of the same background (unpaired Student’s T-test with Welch’s correction). (E-F) Group averages of the percent of time of the total 30-minute test wild-type mice (E) and *Acad10*-deficient mice (F) engaged in resting, ambulating, rearing, and movements while stationary (e.g., grooming). *Acad10*-deficient mice spent a statistically significantly greater percent of time resting and, conversely, statistically significantly smaller percentage of time ambulating, rearing, or engaged in movements while stationary, compared to wild-type mice of the same background (unpaired Student’s T-test with Welch’s correction, p-value <0.05).

ACAD10 has significant expression in mouse brain, but deficient animals showed no overt neurologic symptoms, such as hyperactivity, lethargy, or movement abnormalities visually. To evaluate for potential subtle alterations, neurobehavioral testing was performed on mutant and wild-type mice. Overall, *Acad10-*deficient animals were less active. Ambulatory distance, time spent resting, and number of times rearing occurred each was significantly reduced in *Acad10-*deficient mice compared to wild-type mice (Fig 4A–4C). Despite the significant reduction in ambulatory distance, the speed at which mice traveled did not differ significantly between the genotypes (18.6cm/s ± 1.0 for wild-type mice and 19.3cm/s ± 1.3 for *Acad10*-deficient mice). The tendencies of *Acad10*-deficient mice to be significantly less active and to spend more time resting were also observed when the percent time engaged in the respective behaviors was analyzed and compared (Fig 4E and 4F).

**Fig 5:**
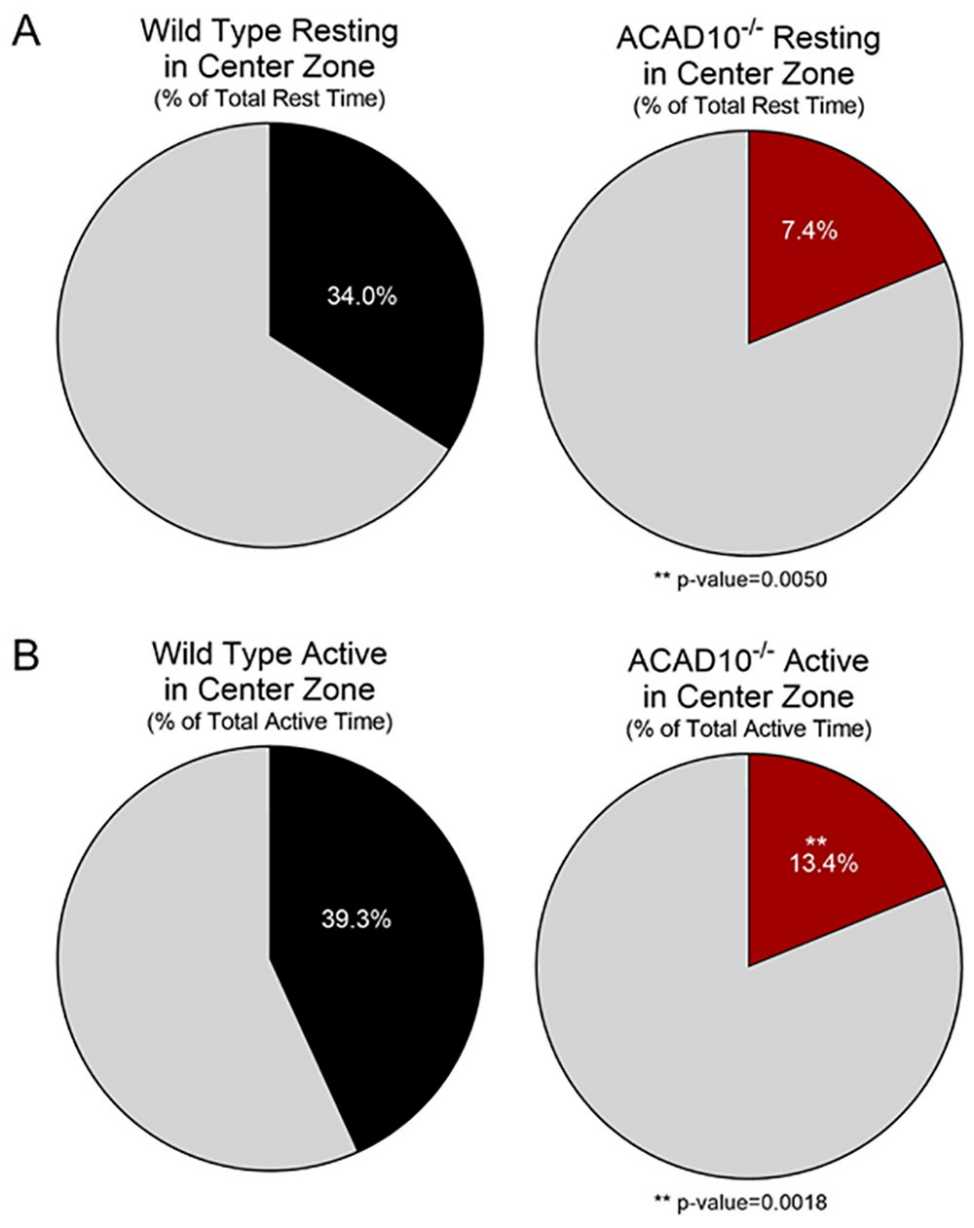
Temporal distribution of activities in the open field test by wildtype and *Acad10-*deficient mice. (A-B) Group averages of the percent of total rest time or percent of total active time spent in the center (colored segments) versus the surround (grey segments) of the the open field arena for wild-type mice (black) and *Acad10-*deficient (red) mice. Of the total amount of time mice rested (A) or were active (i.e., engaged in locomotion, rearing, or movements while stationary; B), the percent of time mice did so in the center of the test arena was statistically significantly lower for *Acad10-*deficient mice compared to wild-type mice of the same background (unpaired Student’s T-test with Welch’s correction, p-value <0.05).

Anxious mice tend to avoid open spaces and preferentially stay close to walls, i.e., they exhibit thigmotaxis [17]. To determine whether *Acad10-*deficient mice differ from wild-type mice in terms of basal anxiety, we calculated for each mouse the amount of time engaged in any of the three active behaviors (ambulation, rearing, movements while stationary) while the mouse was in the center of the test arena relative to the total time the mouse engaged in any of these three activities. Similarly, we calculated for each mouse the percent of rest time while in the center of the arena relative to the mouse’s total rest time. Regardless of whether mice were active or rested, the percent time mice spent engaged in these actions while in the center was significantly lower for *Acad10*-deficient mice than for wild-type mice (Fig 5). In other words, *Acad10-*deficient mice exhibited enhanced thigmotaxis relative to wild-type mice. The *Acad10-*deficient mice’s preference for the safer regions within the open-field chamber, combined with their reduced levels of exploration, whether in the horizontal plane (i.e., ambulation) or the vertical plane (i.e., rearing) suggests that ACAD10 deficiency results in an anxious phenotype.

### DigiGait assessment of gait dynamics

DigiGait testing was used to further characterize subtle changes in motor function through analysis of gait dynamics while mice were walking on a motorized transparent treadmill belt. The more fine-grained analysis of the DigiGait testing revealed no differences between genotypes in any of the gait parameters analyzed, including braking or brake stance, propulsion or propel stance, stride length or stride frequency, swing, or stance (S1 Fig). Based on these analyses, ACAD10 deficiency does not appear to result in any notable impairment of gait (S2 Fig). The markedly reduced ambulatory activity in the open-field tests therefore cannot readily be attributed to impaired motor function in the *Acad10-*deficient mice.

## Discussion

Over the past 30 years, 9 inborn errors of metabolism caused by deficiencies in ACADs have been described. They cause significant morbidity and mortality in affected individuals. Recently, two new ACADs of unknown function, *ACAD10* and *ACAD11*, have been identified in humans through sequencing projects, raising the specter of additional unrecognized disorders related to their dysfunction. *ACAD10* and *ACAD11* share 46% homology and are widely conserved across evolution [4, 18, 19]. Extensive sequence analysis indicates that they are more highly conserved across evolution than the other more highly expressed ACADs, suggesting critical functions for these novel enzymes. Both genes are located within complicated loci containing multiple predicted exons and protein domains, including the ACAD domain. Database searches of transcribed sequences from these genes have identified the presence of multiple transcripts that differed mostly at either the 5’ or 3’ end. *ACAD10* and *ACAD11* transcripts encoding 1059-aa (119 kDa) and 780-aa (87 kDa) proteins predicted to be structurally related have been identified in databases, but their function is unknown. Previous publications on *ACAD10* suggested a novel role in central nervous system metabolism and immunity, and highlighted a possible role in the development of T2DM [4, 20].

Although *Acad10-*deficient mice appeared to be grossly normal neurologically, more focused neurobehavioral testing showed clear abnormalities. They were markedly less active in the open-field testing arena, a characteristic that could potentially be a factor in their tendency to develop obesity. The constellation of reduced exploratory activity without evidence of motor impairment and increased avoidance of open and, thus, more anxiety-inducing spaces suggests that ACAD10 deficiency may be a contributing factor to altered neurological functioning leading to elevated anxiety levels. More detailed analyses of emotional behavior is necessary to confirm these observations. It is interesting that mutant animals exhibited no evidence of gross muscular dysfunction given the previous recognition of fasting-induced rhabdomyolysis, a classic characteristic of long chain fatty acid oxidation muscle disease. Given the prominent role that physiologic stress plays in eliciting symptoms of fatty acid oxidation in other mouse models and human disease, it is conceivable that *Acad10*-deficient mice might exhibit more readily detectable changes in neuromuscular function under stress. Unfortunately, such conditions will also change behavioral performance itself, making variations in mutant animals difficult to interpret. Of note, we have previously shown that directed metabolomics of blood, liver, and muscle extracts of mutant animals did not identify a specific abnormal metabolite. Although we were able to perform directed metabolomic analysis on brain tissue, it seems likely that an undirected metabolomics approach including lipidomics will be necessary to delineate the biochemical derangement caused by ACAD10 deficiency. ACAD10 deficiency has not yet been identified in humans, and our findings leave open the possibility of a wide variety of phenotypes including fasting or stress induce rhabdomyolysis, obesity, and type 2 diabetes mellitus, and primarily neurologic symptoms.

In summary, we have shown that *Acad10*-deficient mice have normal brain structure and histology. However, they exhibit a subtle neurobehavioral phenotype consistent with expression of the gene in fetal and adult brains. These findings add to the potential phenotypes of ACAD10 deficiency in humans.

## Supporting information

S1 Raw Images (PDF)

S1 Fig DigiGait indices. (A-H) Group averages (horizontal lines) of brake, brake stance, propel, propel stance, stride length, stride frequency, swing, and stance for wildtype (black dots) and *Acad10-*deficient mice (red dots). No statistically significant differences between genotypes were observed (unpaired parametric T-test with Welch’s correction; all p-values > 0.1).
(TIF)

S2 Fig Gait dynamic. Qualitative shape and quantitative timing of gait signals obtained by the DigiGait system for the 4 limbs throughout ~9 strides from representative wild-type and mutant animals.
(TIF)

S1 Table Acylcarnitine levels in wild-type and *Acad10-*deficient mouse brain and plasma. Table shows means ± standard deviation.
(DOCX)
