# Supplementary figures and images for: ACAD10 protein expression and Neurobehavioral assessment of Acad10-deficient mice
Source: PLoS One. 2020 Dec 10;15(12):e0242445. doi: 10.1371/journal.pone.0242445 (PMC7728233; doi:10.1371/journal.pone.0242445)

Figure 1. Original images

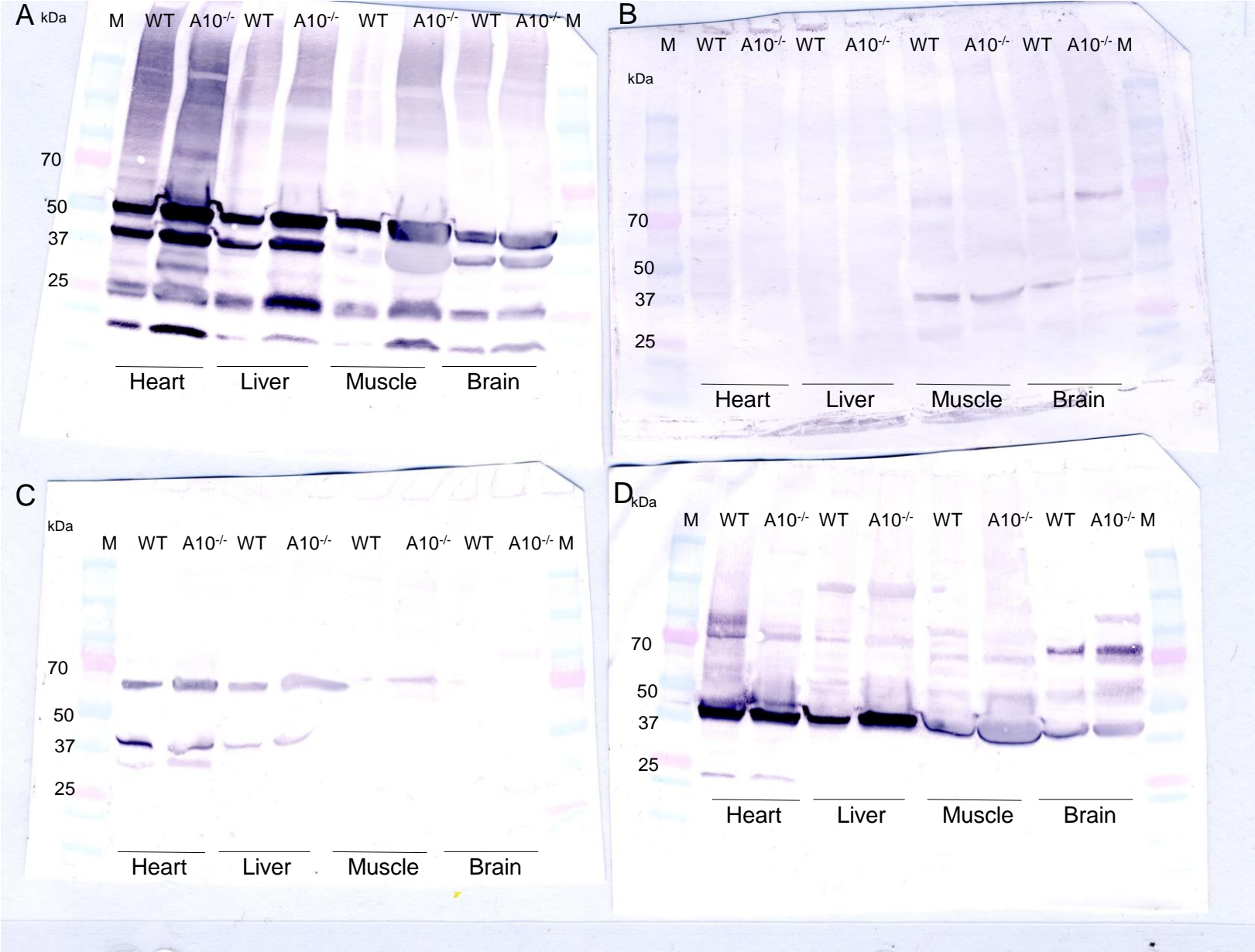

Supplement: S1 Raw Images — (PDF) [file pone.0242445.s001.pdf]

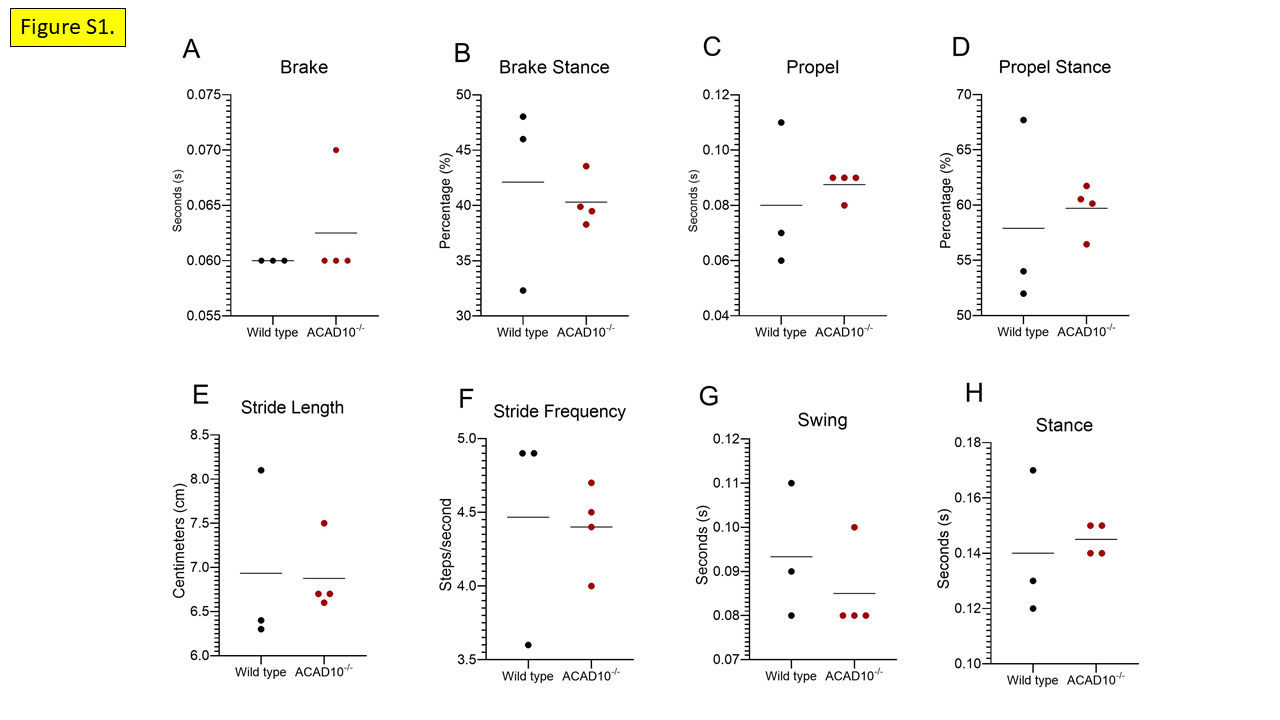

Supplement: S1 Fig — (A-H) Group averages (horizontal lines) of brake, brake stance, propel, propel stance, stride length, stride frequency, swing, and stance for wildtype (black dots) and Acad10-deficient mice (red dots). No statistically significant differences between genotypes were observed (unpaired parametric T-test with Welch’s correction; all p-values > 0.1). (TIF) [file pone.0242445.s002.tif]

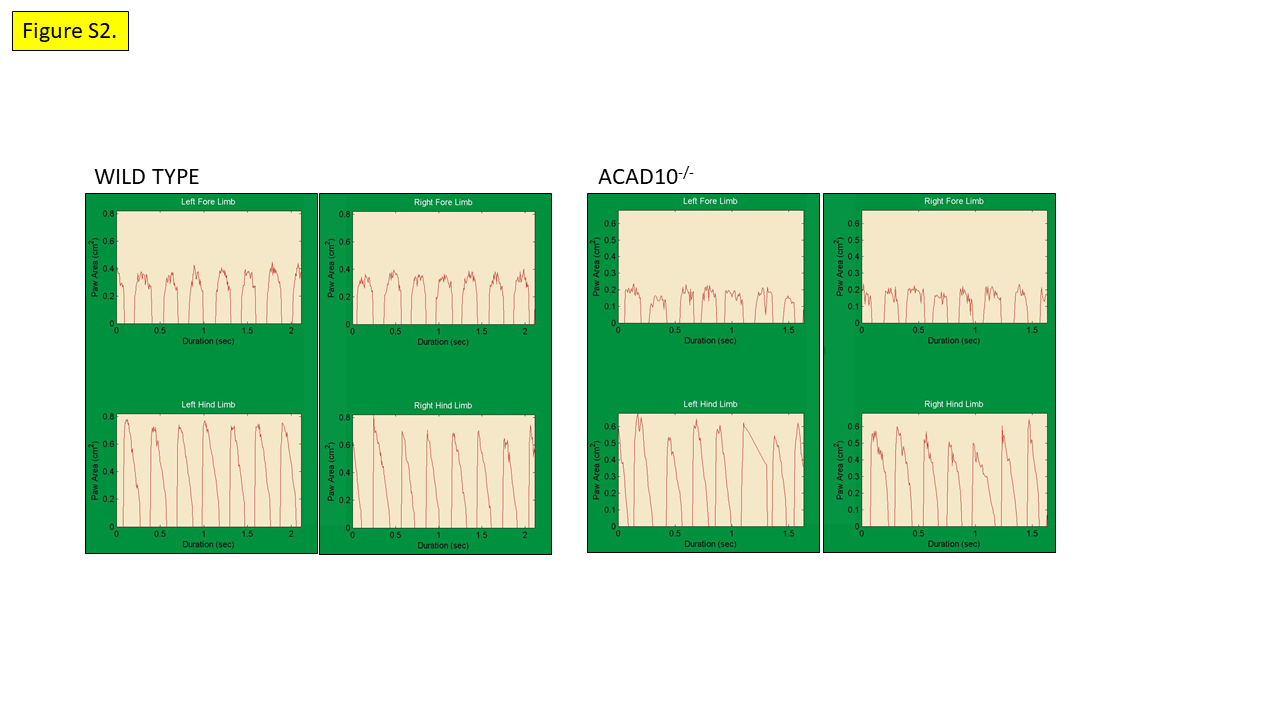

Supplement: S2 Fig — Qualitative shape and quantitative timing of gait signals obtained by the DigiGait system for the 4 limbs throughout ~9 strides from representative wild-type and mutant animals. (TIF) [file pone.0242445.s003.tif]
