# Supplementary material for: ACAD10 protein expression and Neurobehavioral assessment of Acad10-deficient mice
Source: PLoS One. 2020 Dec 10;15(12):e0242445. doi: 10.1371/journal.pone.0242445 (PMC7728233; doi:10.1371/journal.pone.0242445)
Supplement: S1 Table — Table shows means ± standard deviation. (DOCX) [file pone.0242445.s004.docx]

|  | Brain | | Plasma | |
| --- | --- | --- | --- | --- |
| Acylcarnitine species | Wild-type (mean ± SD) | ACAD10-/- (mean ± SD) | Wild-type (mean ± SD) | ACAD10-/- (mean ± SD) |
| acetylcarnitine | 56.845 ± 6.66 | 45.831 ± 14.37 | 20.291 ± 5.17 | 19.51 ± 0.01 |
| propionylcarnitine | 3.061 ± 1.23 | 2.554 ± 1.16 | 0.810 ± 0.59 | 0.700 ± 0.01 |
| butyrylcarnitine | 1.327 ± 0.20 | 1.323 ± 0.27 | 0.581 ± 0.30 | 0.599 ± 0.01 |
| isobutyrylcarnitine | 0.438 ± 0.08 | 0.346 ± 0.17 | 0.124 ± 0.06 | 0.136 ± 0.01 |
| R-3-hydroxy-butyrylcarnitine | 0.719 ± 0.53 | 0.633 ± 0.50 | 0.047 ± 0.01 | 0.044 ± 0.01 |
| S-3-hydroxy-butyrylcarnitine | 1.076 ± 0.68 | 0.974 ± 0.61 | 0.069 ± 0.02 | 0.102 ± 0.01 |
| valerylcarnitine | 0.148 ± 0.11 | 0.163 ± 0.15 | 0.008 ± 0.01 | 0.016 ± 0.01 |
| isovalerylcarnitine | 0.644 ± 0.22 | 0.789 ± 0.62 | 0.107 ± 0.06 | 0.13 ± 0.01 |
| 3-hydroxy-isovalerylcarnitine | 0.945 ± 0.51 | 0.902 ± 0.43 | 0.08 ± 0.01 | 0.074 ± 0.01 |
| 2-methyl-butyrylcarnitine | 0.570 ± 0.20 | 0.534 ± 0.24 | 0.085 ± 0.03 | 0.091 ± 0.01 |
| tigloylcarnitine | 0.149 ± 0.13 | 0.137 ± 0.12 | 0.009 ± 0.00 | 0.008 ± 0.01 |
| 3-methyl-crotonylcarnitine | 0.024 ± 0.02 | 0.017 ± 0.01 | 0 ± 0.00 | 0 ± 0.01 |
| hexanoylcarnitine | 0.209 ± 0.13 | 0.203 ± 0.15 | 0.037 ± 0.02 | 0.046 ± 0.01 |
| R-3-hydroxy-hexanoylcarnitine | 0.083 ± 0.06 | 0.068 ± 0.04 | 0.001 ± 0.00 | 0.001 ± 0.01 |
| S-3-hydroxy-hexanoylcarnitine | 0.116 ± 0.12 | 0.125 ± 0.14 | 0.001 ± 0.00 | 0.005 ± 0.01 |
| phenylacetylcarnitine | 0.07 ± 0.11 | 0.08 ± 0.13 | 0.004 ± 0.01 | 0.003 ± 0.01 |
| phenylpropionylcarnitine | 0.26 ± 0.10 | 0.24 ± 0.07 | 0.01 ± 0.00 | 0.02 ± 0.01 |
| 4-phenyl-butyrylcarnitine | 0.160 ± 0.13 | 0.155 ± 0.13 | 0.005 ± 0.01 | 0.005 ± 0.01 |
| benzoylcarnitine | 0.013 ± 0.02 | 0.014 ± 0.01 | 0.003 ± 0.01 | 0.001 ± 0.01 |
| 4-methyl-hexanoylcarnitine | 0.14 ± 0.12 | 0.14 ± 0.12 | 0.007 ± 0.01 | 0.007 ± 0.01 |
| octanoylcarnitine | 0.233 ± 0.21 | 0.22 ± 0.19 | 0.013 ± 0.01 | 0.013 ± 0.01 |
| R-3-hydroxy-octanoylcarnitine | 0.158 ± 0.14 | 0.204 ± 0.18 | 0.007 ± 0.00 | 0.008 ± 0.01 |
| S-3-hydroxy-octanoylcarnitine | 0.243 ± 0.20 | 0.285 ± 0.25 | 0.016 ± 0.01 | 0.016 ± 0.01 |
| branched-chain C8 | 0.117 ± 0.10 | 0.11 ± 0.10 | 0.007 ± 0.01 | 0.007 ± 0.01 |
| cis-3 4-methylene-heptanoylcarnitine | 0.198 ± 0.17 | 0.197 ± 0.17 | 0.007 ± 0.01 | 0.007 ± 0.01 |
| 4-methyl-octanoylcarnitine | 0.237 ± 0.23 | 0.219 ± 0.21 | 0.01 ± 0.01 | 0.01 ± 0.01 |
| 2 6-dimethyl-heptanoylcarnitine | 0.284 ± 0.25 | 0.287 ± 0.26 | 0.01 ± 0.01 | 0.01 ± 0.01 |
| decanoylcarnitine | 0.088 ± 0.10 | 0.08 ± 0.10 | 0.009 ± 0.01 | 0.010 ± 0.01 |
| cis-4-decenoylcarnitine | 0.036 ± 0.05 | 0.080 ± 0.13 | 0.008 ± 0.01 | 0.009 ± 0.01 |
| cis-3 4-methylene-nonanoylcarnitine | 0.020 ± 0.03 | 0.04 ± 0.07 | 0.003 ± 0.01 | 0.004 ± 0.01 |
| R-3-hydroxy-decanoylcarnitine | 0.115 ± 0.15 | 0.1 ± 0.12 | 0.007 ± 0.00 | 0.008 ± 0.01 |
| S-3-hydroxy-decanoylcarnitine | 0.420 ± 0.36 | 0.381 ± 0.33 | 0.025 ± 0.02 | 0.016 ± 0.01 |
| 5-decynoylcarnitine | 0.083 ± 0.08 | 0.077 ± 0.08 | 0.003 ± 0.01 | 0.003 ± 0.01 |
| lauroylcarnitine | 0.302 ± 0.13 | 0.21 ± 0.14 | 0.019 ± 0.01 | 0.019 ± 0.01 |
| trans-2-dodecenoylcarnitine | 0.093 ± 0.13 | 0.094 ± 0.11 | 0.007 ± 0.01 | 0.004 ± 0.01 |
| R-3-hydroxy-lauroylcarnitine | 0.14 ± 0.15 | 0.143 ± 0.16 | 0.004 ± 0.01 | 0.004 ± 0.01 |
| S-3-hydroxy-lauroylcarnitine | 0.214 ± 0.21 | 0.074 ± 0.10 | 0.003 ± 0.01 | 0.004 ± 0.01 |
| myristoylcarnitine | 1.366 ± 0.50 | 0.917 ± 0.13 | 0.036 ± 0.01 | 0.031 ± 0.01 |
| myristoleoylcarnitine | 0.403 ± 0.17 | 0.389 ± 0.23 | 0.018 ± 0.01 | 0.019 ± 0.01 |
| cis-5-tetradecenoylcarnitine | 0.087 ± 0.15 | 0.063 ± 0.11 | 0.020 ± 0.02 | 0.019 ± 0.01 |
| trans-2-tetradecenoylcarnitine | 0.199 ± 0.29 | 0.23 ± 0.23 | 0.004 ± 0.01 | 0.007 ± 0.01 |
| cis cis-5 8-tetradecadienoylcarnitine | 0.192 ± 0.27 | 0.17 ± 0.23 | 0.020 ± 0.01 | 0.017 ± 0.01 |
| R-3-hydroxy-myristoylcarnitine | 0.12 ± 0.14 | 0.113 ± 0.12 | 0.003 ± 0.01 | 0.007 ± 0.01 |
| S-3-hydroxy-myristoylcarnitine | 0.083 ± 0.13 | 0.09 ± 0.14 | 0.004 ± 0.01 | 0.007 ± 0.01 |
| hydroxy-C14:1 | 0.077 ± 0.12 | 0.072 ± 0.11 | 0.008 ± 0.00 | 0.008 ± 0.01 |
| palmitoylcarnitine | 3.207 ± 0.82 | 2.555 ± 0.71 | 0.126 ± 0.02 | 0.111 ± 0.01 |
| palmitoleoylcarnitine | 0.44 ± 0.40 | 0.306 ± 0.29 | 0.016 ± 0.01 | 0.019 ± 0.01 |
| trans-2-hexadecenoylcarnitine | 0.172 ± 0.22 | 0.207 ± 0.24 | 0.004 ± 0.01 | 0.004 ± 0.01 |
| R-3-hydroxy-palmitoylcarnitine | 0.313 ± 0.29 | 0.3 ± 0.27 | 0.014 ± 0.01 | 0.013 ± 0.01 |
| S-3-hydroxy-palmitoylcarnitine | 0.119 ± 0.20 | 0.107 ± 0.18 | 0.005 ± 0.01 | 0.005 ± 0.01 |
| hydroxy-C16:1 | 0.126 ± 0.19 | 0.118 ± 0.20 | 0.007 ± 0.01 | 0.004 ± 0.01 |
| stearoylcarnitine | 1.129 ± 0.83 | 1.327 ± 0.87 | 0.052 ± 0.02 | 0.047 ± 0.01 |
| oleoylcarnitine | 2.189 ± 0.75 | 1.903 ± 0.95 | 0.082 ± 0.03 | 0.081 ± 0.01 |
| linoleoylcarnitine | 0.525 ± 0.34 | 0.397 ± 0.36 | 0.051 ± 0.02 | 0.052 ± 0.01 |
| alpha-linolenoylcarnitine | 0.157 ± 0.14 | 0.087 ± 0.15 | 0.008 ± 0.00 | 0.009 ± 0.01 |
| gamma-linolenoylcarnitine | 0.114 ± 0.18 | 0.12 ± 0.21 | 0.004 ± 0.01 | 0.007 ± 0.01 |
| R-3-hydroxy-stearoylcarnitine | 0.300 ± 0.28 | 0.263 ± 0.26 | 0.018 ± 0.01 | 0.012 ± 0.01 |
| S-3-hydroxy-stearoylcarnitine | 0.144 ± 0.13 | 0.13 ± 0.11 | 0.007 ± 0.01 | 0.007 ± 0.01 |
| hydroxy-C18:1 | 0.169 ± 0.12 | 0.192 ± 0.12 | 0.008 ± 0.00 | 0.009 ± 0.01 |
| hydroxy-C18:2 | 0.128 ± 0.11 | 0.135 ± 0.11 | 0.008 ± 0.00 | 0.008 ± 0.01 |
| hydroxy-C18:3 | 0.137 ± 0.11 | 0.13 ± 0.12 | 0.007 ± 0.00 | 0.007 ± 0.01 |
| malonylcarnitine | 0.343 ± 0.41 | 0.41 ± 0.14 | 0.029 ± 0.01 | 0.016 ± 0.01 |
| succinylcarnitine | 0.283 ± 0.13 | 0.299 ± 0.03 | 0.024 ± 0.01 | 0.032 ± 0.01 |
| methyl-malonylcarnitine | 0.156 ± 0.21 | 0.15 ± 0.17 | 0.015 ± 0.01 | 0.015 ± 0.01 |
| ethyl-malonylcarnitine | 0.08 ± 0.07 | 0.08 ± 0.08 | 0.003 ± 0.01 | 0.003 ± 0.01 |
| glutaroylcarnitine | 0.473 ± 0.22 | 0.286 ± 0.22 | 0.008 ± 0.01 | 0.020 ± 0.01 |
| adipoylcarnitine | 0.213 ± 0.17 | 0.176 ± 0.15 | 0.022 ± 0.01 | 0.02 ± 0.01 |
| 3-methyl-glutaroylcarnitine | 0.12 ± 0.20 | 0.103 ± 0.18 | 0.007 ± 0.01 | 0.003 ± 0.01 |
| suberoylcarnitine | 0.14 ± 0.19 | 0.123 ± 0.16 | 0.017 ± 0.01 | 0.013 ± 0.01 |
| sebacoylcarnitine | 0.12 ± 0.11 | 0.127 ± 0.11 | 0.007 ± 0.00 | 0.007 ± 0.01 |
